# Concordance of the Indian Mental Healthcare Act 2017 with the World Health Organization’s Checklist on Mental Health Legislation

**DOI:** 10.1186/s13033-017-0155-1

**Published:** 2017-08-18

**Authors:** Richard M. Duffy, Brendan D. Kelly

**Affiliations:** Department of Psychiatry, Trinity College Dublin, Trinity Centre for Health Science, Tallaght Hospital, Dublin, D24 NR0A Ireland

**Keywords:** Human rights, Jurisprudence, Psychiatry, Mental disorders, Legislation, Coercion

## Abstract

**Background:**

India is revising its mental health legislation with the Indian Mental Healthcare Act 2017 (IMHA). When implemented, this legislation will apply to over 1.25 billion people. In 2005, the World Health Organization (WHO) published a Resource Book (WHO-RB) on mental health, human rights and legislation, including a checklist of 175 specific items to be addressed in mental health legislation or policy in individual countries. Even following the publication of the United Nations Convention on the Rights of Persons with Disabilities (CRPD) (2006), the WHO-RB remains the most comprehensive checklist for mental health legislation available, rooted in UN and WHO documents and providing the most systematic, detailed framework for human rights analysis of mental health legislation. We sought to determine the extent to which the IMHA will bring Indian legislation in line with the WHO-RB.

**Methods:**

The IMHA and other relevant pieces of Indian legislation are compared to each of the items in the WHO-RB. We classify each item in a binary manner, as either concordant or not, and provide more nuanced detail in the text.

**Results:**

The IMHA addresses 96/175 (55.4%) of the WHO-RB standards examined. When other relevant Indian legislation is taken into account, 118/175 (68.0%) of the standards are addressed in Indian law. Important areas of low concordance include the rights of families and carers, competence and guardianship, non-protesting patients and involuntary community treatment. The important legal constructs of advance directives, supported decision-making and nominated representatives are articulated in the Indian legislation and explored in this paper.

**Conclusions:**

In theory, the IMHA is a highly progressive piece of legislation, especially when compared to legislation in other jurisdictions subject to similar analysis. Along with the Indian Rights of Persons with Disabilities Act 2016, it will bring Indian law closely in line with the WHO-RB. Vague, opaque language is however, used in certain contentious areas; this may represent arrangement-focused rather than realisation-focused legislation, and lead to inadvertent limitation of certain rights. Finally, the WHO-RB checklist is an extremely useful tool for this kind of analysis; we recommend it is updated to reflect the CRPD and other relevant developments.

## Background

### Mental health in India

Standing at over 1.25 billion, the population of India is the second largest in the world, behind only China. The United Nations (UN) predicts that by 2022 India’s population will surpass that of China and by 2030 India’s population will reach 1.5 billion [1].

Mental health is a major concern in India; major depressive disorder is the leading cause of years lived with disability and anxiety is the ninth leading cause [2]. It is estimated that just over one in ten people in India have a mental health issue, one in twenty people suffer from depression, and 0.8% have a “*common and severe mental disorder*” [3]. The number of individuals affected by mental illness is enormous; it is estimated that 2.5 million people have schizophrenia, 8.8 million have bipolar affective disorder (BPAD), 36.8 million have anxiety disorders and 13.4 million have alcohol dependence [4]. In 2013, just under 31 million disability-adjusted-life-years (DALY) were due to mental, neurological and substance misuse disorders. Schizophrenia accounted for 1.7 million of those, BPAD for 1.8 million, depression for 11.5 million, alcohol and substance misuse for 3 million, and dementia for 1.8 million [5]. Males in the 30–49 age group have the highest prevalence of mental morbidity; in addition to the impact on these individuals and their families, this has major implications for India’s productivity [3].

Despite the large burden of mental illness only 10% of Indians with mental health problems receive evidence-based treatments [6]. Treatment gaps greater than 70% exist due to insufficient funding of mental, neurological, and substance use disorders [3, 5]. India’s spending on mental health care has consistently been inadequate [7]. In 2011, India spent 4.16% of its gross domestic product on health; 0.06% of this was allocated at a national level for outpatient psychiatric care [8]. India’s number of mental health beds is well below average with only 2.15 beds per 100,000 compared to the global figure of 6.5 [7].

As the burden of mental illness is increasingly recognized, funding is being increased with the hope of ensuring more people receive high quality health care. India is implementing a variety of initiatives to address this large need, close the treatment gap, and reduce the DALYs lost to mental, neurological and substance misuse disorders [9]. These initiatives need to be supported by clear, pragmatic and robust mental health law in line with international human rights legislation.

Mental health legislation is an essential part of delivering high quality mental health care and is especially necessary to protect the rights of individuals receiving such care. At present many countries lack appropriate mental health legislation and consequently many individuals are deprived safe, effective, person-centred services. This has a significant impact on occupational, personal and family life [10]. India has previously led the way in the developing world in attempting to shift the care of individuals with mental illness from asylums to community-based treatments [11], however, without clear legislation and policies and a lack of community based services, results were less than satisfactory [12].

India now leads the way globally in revising mental health legislation in line with international human rights standards. It is hoped that on this occasion that the desired mental health service will be realised through appropriate legislation and implementation. The WHO is encouraging countries to update their mental health legislation in line with international guidelines and hopes that 50% of countries will achieve this by 2020 [10]. With so many countries needing to revise their laws concerning mental health, India’s proposed revision and its implementation will be highly relevant to many other countries, especially those who have also ratified the UN-Convention on the Rights of Persons with Disabilities (UN-CRPD).

### The UN-Convention on the Rights of Persons with Disabilities (UN-CRPD)

In 2006, the UN-CRPD was published and it came into force in 2008. Since then it has been signed by over 160 countries [13]. India ratified the UN-CRPD in 2007 [14].

Under the UN-CRPD, persons with disabilities include those with long-term mental or intellectual impairment [15, 16]. The UN-CRPD attempts to emphasize and address the attitudinal and environmental barriers that individuals with impairments face. This has been perceived as a progressive and irreversible step away from a “medical model” of disability and towards a social model. Rao et al., describe it as a move from a “charity based” to a “rights based” approach to disability [17].

The UN-CRPD appears strongly opposed to involuntary treatments [18] and affirms the legal capacity of individuals at all times. The convention requires that ratifying countries revise their laws to make them concordant with the convention. Consequently, India’s mental health care legislation needed to be reformed and the UN-CRPD duly prompted the drafting of two important pieces of legislation in India: the Mental Healthcare Act 2017 (IMHA) and the Rights of Persons with Disability Act 2016 (RPDA) [17].

The drafting of the UN-CRPD was a long and complex process. In contrast to prior international human rights treaties, human rights organisations were heavily involved from the outset [19]. The World Network of Users and Survivors of Psychiatry played a highly influential role and set forth its views on capacity as non-negotiable, it sought to ban institutional care and forced treatment [20]. Much debate occurred concerning emergency circumstances but in the end time ran out and no provisions were made for these [21]. This may call into question the UN-CRPDs ability to address all mental health issues, in particular emergency situations. Currently, however, the UN-CRPD provides the legal framework for mental health legislation in all countries that have ratified it.

### India’s mental health legislation

The first mental health legislation in India was introduced by the British colonial government in 1858, when three Acts relating to mental health were adopted: the Lunacy (Supreme Courts) Act, the Lunacy (District Courts) Act and the Indian Lunatic Asylum Act [22]. These acts focused on asylum-based care but, due to the conditions that many patients found themselves in, pressure mounted on government to reform mental healthcare more generally. In 1912, the Indian Lunacy Act was passed.

Following Indian independence, the Indian Psychiatric Society submitted a revised mental healthcare Bill in 1950 which was finally enacted as the Mental Health Act in 1987. This document introduced many important changes, including modern terminology, the creation of the Central and State mental health authorities, prohibition of nonconsensual research, and simplification of discharge procedures [23]. The 1987 legislation, however, faced a lot of criticism from the outset [24]: concerns were raised that it gave more emphasis to legal consideration rather than medical care; its position on the family was criticized; and it failed to make provisions for home-based treatments, among other matters [23]. From the perspective of international law, moreover, the 1987 legislation was not in line with the UN-CRPD when it was published in 2006.

Consequently, India has recently revised its mental health legislation with a new law that has been greatly anticipated [25–28]. On the 8 August 2016 the Rajya Sabha (the upper house of the Indian parliament) unanimously passed The Mental Healthcare Bill, 2016. The stated aim of the Bill was “to provide for mental healthcare and services for persons with mental illness and to protect, promote and fulfil the rights of such persons during delivery of mental healthcare and services and for matters connected therewith or incidental thereto.” This has now been adopted as the IMHA which received the assent of the President on 7 April, 2017.

### The Rights of Persons with Disabilities Act 2016 (RPDA)

The IMHA is not the only significant legislative reform in this area in India in recent years; in 2016 the RPDA replaced the Persons with Disability Act 1995. The RPDA received the assent of the Indian President on 27 December 2016 and like the IMHA it explicitly states that its purpose is to give effect to the UN-CRPD.

The RPDA complements the proposed IMHA and legally underpins many of the social and economic rights of individuals with mental illness. In particular, it emphasises respect for inherent dignity, individual autonomy including the freedom to make one’s own choices, and independence of persons; non-discrimination; full and effective participation and inclusion in society; respect for difference and acceptance of persons with disabilities as part of human diversity and humanity; equality of opportunity; accessibility; equality between men and women; respect for the evolving capacities of children with disabilities and respect for the right of children with disabilities to preserve their identities. More specific provisions of relevance to mental illness are discussed further in the relevant sections of this paper.

Two main concerns have been raised about the RPDA. First, it appears to lack synchronicity with the IMHA in certain important respects (e.g.it is not clear how guardianship and nominated representatives will be related). Second, it is questionable whether the general nature of the RPDA enables it to address the particular challenges presented by mental illness [18]. This concern is underlined by the fact that the IMHA does not directly address many of the areas of discrimination or social rights highlighted in the UN-CRPD or the WHO *Resource Book on Mental Health, Human Rights and Legislation* (WHO-RB) (below). These matters are of considerable relevance to the analysis presented in this paper, which considers the new Indian legislation in the context of the standards set out in the WHO-RB, and they are discussed in the relevant sections of the paper.

### The WHO Resource Book on Mental Health, Human Rights and Legislation (WHO-RB)

Published in 2005, the WHO-RB seeks to provide guidance to governments on the development of human rights-centred mental health legislation. The largest single section of the document identifies and discusses the key legal issues that should be addressed in national mental health legislation or policy, summarised in Annex One as the “Checklist on mental health legislation”. The WHO checklist contains 175 items, divided into 27 sections, covering all key areas of mental health law.

The checklist, although explicitly informed by the Universal Declaration of Human Rights [29], is not a set of absolute rules and it is not legally binding. There are no sanctions for states which fail to accord with its standards and, unlike the UN International Covenant on Civil and Political Rights, the UN Human Rights Committee does not review WHO member states’ reports on compliance. The WHO-RB checklist is, instead, designed to work by influencing states as they redraft and implement national mental health laws. Given the checklist’s close links with the Universal Declaration of Human Rights, the authors make the assumption that its standards will be accepted by the international community and deemed worth reflecting in national mental health law [30].

It is arguable, however, that some of the issues that the WHO suggests should be covered by mental health legislation should be covered by mental health, public health or social policy instead. Indeed, the WHO explicitly states that some countries may address some or all of these issues in general legislation (e.g. equality legislation), other forms of (not legally-binding) regulation, or mental health policy, rather than in specific mental health legislation [30]. The history of psychiatry, however, supports the particular importance of dedicated mental health legislation, rather than non-binding regulation or general law, for protecting the rights of the mentally-ill. The WHO also acknowledges the centrality of law in this process by presenting its final checklist in the WHO-RB as a “Checklist on mental health *legislation*” (our italics). This is why the WHO-RB checklist forms the focus of this analysis: it is the most detailed and comprehensive human rights-based framework developed to date for the analysis of national mental health legislation.

The WHO-RB has two serious limitations and is not the unquestionable gold standard for mental health legislation. Most significantly, it was drafted before the UN-CRPD was completed and consequently is at odds with it in some areas [31]. It discusses involuntary treatment, loss of capacity and emergency treatments these directly conflict with the UN-CRPD. The WHO-RB also does not have the legal standing that the UN-CRPD has.

Ofori-Atta et al. have previously used the WHO Checklist to inform their evaluation of mental health legislation in Ghana [32]. A more quantitative, formal approach was adopted by Kelly who compared166 checklist items with English, Welsh and Irish mental health legislation [30, 33]. Kelly found that the Mental Health Act 2007 in England and Wales met 54.2% of the WHO standards while the Irish Mental Health Act 2001 met 42.2%. Both Mental Health Acts were found to inadequately address fundamental principles particularly in relation to the rights of voluntary patients, vulnerable patient groups, emergency treatments and economic and social rights.

In the present paper, we adapt a similar methodological approach to the new Indian legislation, seeking to identify the extent to which the IMHA brings Indian legislation into line with the WHO-RB, a key document in this field which also overlaps to a significant (but incomplete) extent with the standards outlined in the UN-CRPD (above).

## Methods

This study adopts a “black letter” approach, similar to that used by Kelly [30, 33]. In such an analysis, the focus is on the *content* of the legislation rather than its *effect*. Therefore, this paper primarily compares the written content of the IMHA to the WHO-RB’s checklist (175 items). Where relevant, other pieces of Indian legislation are also considered; e.g. the RPDA 2016, the Indian Penal Code 1860, the Code of Criminal Procedure 1973, the Medical Termination of Pregnancy Act 1971, and the Narcotic Drugs and Psychotropic Substances Act 1985. In order to draw useful information from the results, we classify each WHO standard as either being met or not met in Indian legislation. Where there is an element of uncertainty, we continue with this dichotomous classification system but discuss the particular item in more detail in the text.

The WHO-RB’s checklist is divided into 27 sections, each identified by a capital letter. Specific standards contained in each section are further identified by numbers, lower case letters and Roman numerals. For clarity and to assist with navigation, these have been included in parentheses in the text.

Two specific methodological points merit mention here. First, emergency treatments laid out in the IMHA are not considered as “involuntary treatments” in our analysis; they are instead compared to the WHO-RB guidelines on “emergency treatments”. Second, section J of the WHO-RB considers “involuntary treatment (when separate from involuntary admission)”; under the IMHA supported (involuntary) treatment is not directly considered outside of a supported (involuntary) admission. However, there is a possibility that in the context of an advance directive a person could receive involuntary treatment outside the context of a supported admission and so we have retained this section and discussed this further in the paper.

## Results

Table 1 lists the WHO standards and identifies which standards are met in the new Indian legislation and which are not. Overall, 55.4% (97/175) of the WHO standards are met directly in the IMHA while 68.0% (119/175) are addressed somewhere in Indian legislation (including both the IMHA and other pieces of legislation). The RPDA is the main piece of legislation outside of the IMHA which addresses specific items of the WHO-RB.

**Table 1:** Concordance of India’s Mental Healthcare Act 2017 with the World Health Organization’s “Checklist on mental health legislation” (World Health Organization, 2005)

Legislative issue				Is Indian legislation concordant or not?
A	Preamble and objectives	1a	Does the legislation have a preamble which emphasises the human rights of people with mental disorders?	Yes
		1b	Does the legislation have a preamble which emphasises the importance of accessible mental health services for all?	Yes
		2a	Does the legislation specify that the purpose and objectives to be achieved include non-discrimination against people with mental disorders?	No
		2b	Does the legislation specify that the purpose and objectives to be achieved include promotion and protection of the rights of people with mental disorders?	Yes
		2c	Does the legislation specify that the purpose and objectives to be achieved include improved access to mental health services?	Yes
		2d	Does the legislation specify that the purpose and objectives to be achieved include a community-based approach?	Yes
B	Definitions	1	Is there a clear definition of mental disorder/mental illness/mental disability/mental incapacity?	Yes
		2	Is it evident from the legislation why the particular term (above) has been chosen?	No
		3	Is the legislation clear on whether or not mental retardation/intellectual disability, personality disorders and substance abuse are being covered in the legislation?	No
		4	Are all key terms in the legislation clearly defined?	Yes
		5	Are all the key terms used consistently throughout the legislation (i.e. not interchanged with other terms with similar meanings)?	Yes
		6	Are all “interpretable” terms (i.e. terms that may have several possible interpretations or meanings or may be ambiguous in terms of their meaning) in the legislation defined?	Yes
C	Access to mental health care	1	Does the legislation make provision for the financing of mental health services?	No
		2	Does the legislation state that mental health services should be provided on an equal basis with physical health care?	Yes
		3	Does the legislation ensure allocation of resources to underserved populations and specify that these services should be culturally appropriate?	No
		4	Does the legislation promote mental health within primary health care?	Yes
		5	Does the legislation promote access to psychotropic drugs?	Yes
		6	Does the legislation promote a psychosocial, rehabilitative approach?	Yes
		7	Does the legislation promote access to health insurance in the private and public health sector for people with mental disorders?	Yes
		8	Does the legislation promote community care and deinstitutionalisation?	Yes
D	Rights of users of mental health services	1	Does the legislation include the rights to respect, dignity and to be treated in a humane way?	Yes
		2	Is the right to patients’ confidentiality regarding information about themselves, their illness and treatment included?	Yes
		2a	Are there sanctions and penalties for people who contravene patients’ confidentiality?	Yes
		2b	Does the legislation lay down exceptional circumstances when confidentiality may be legally breached?	Yes
		2c	Does the legislation allow patients and their personal representatives the right to ask for judicial review of, or appeal against, decisions to release information?	No
		3	Does the legislation provide patients free and full access to information about themselves (including access to their clinical records)?	Yes
		3a	Are circumstances in which such access can be denied outlined?	Yes
		3b	Does the legislation allow patients and their personal representatives the right to ask for judicial review of, or appeal against, decisions to withhold information?	Yes
		4	Does the law specify the right to be protected from cruel, inhuman and degrading treatment?	Yes
		5	Does the legislation set out the minimal conditions to be maintained in mental health facilities for a safe, therapeutic and hygienic environment?	No
		6	Does the law insist on the privacy of people with mental disorders?	Yes
		6a	Is the law clear on minimal levels of privacy to be respected?	No
		7	Does the legislation outlaw forced or inadequately remunerated labour within mental health institutions?	Yes
		8	Does the law make provision for educational activities; vocational training; leisure and recreational activities; and religious or cultural needs of people with mental disorders?	Yes
		9	Are the health authorities compelled by the law to inform patients of their rights?	Yes
		10	Does legislation ensure that users of mental health services are involved in mental health policy, legislation development and service planning?	Yes
E	Rights of families or other carers	1	Does the law entitle families or other primary carers to information about the person with a mental disorder (unless the patient refuses the divulging of such information)?	No
		2	Are family members or other primary carers encouraged to become involved in the formulation and implementation of the patient’s individualised treatment plan?	No
		3	Do families or other primary carers have the right to appeal involuntary admission and treatment decisions?	No
		4	Do families or other primary carers have the right to apply for the discharge of mentally ill offenders?	No
		5	Does legislation ensure that family members or other carers are involved in the development of mental health policy, legislation and service planning?	Yes
F	Competence, capacity and guardianship	1	Does legislation make provision for the management of the affairs of people with mental disorders if they are unable to do so?	Yes
		2	Does the law define “competence” and “capacity”?	Yes
		3	Does the law lay down a procedure and criteria for determining a person’s incapacity/incompetence with respect to issues such as treatment decisions, selection of a substitute decision-maker, making financial decisions?	Yes
		4	Are procedures laid down for appeals against decisions of incapacity/incompetence, and for periodic reviews of decisions?	No
		5	Does the law lay down procedures for the appointment, duration, duties and responsibilities of a guardian to act on behalf of a patient?	No
		6	Does the law determine a process for establishing in which areas a guardian may take decisions on behalf of a patient?	No
		7	Does the law make provision for a systematic review of the need for a guardian?	No
		8	Does the law make provision for a patient to appeal against the appointment of a guardian?	Yes
G	Voluntary admission and treatment	1	Does the law promote voluntary admission and treatment as a preferred alternative to involuntary admission and treatment?	Yes
		2	Does the law state that all voluntary patients can only be treated after obtaining informed consent?	Yes
		3	Does the law state that people admitted as voluntary mental health users should be cared for in a way that is equitable with patients with physical health problems?	Yes
		4	Does the law state that voluntary admission and treatment also implies the right to voluntary discharge/refusal of treatment?	Yes
		5	Does the law state that voluntary patients should be informed at the time of admission that they may only be denied the right to leave if they meet the conditions for involuntary care?	No
H	Non-protesting patients	1	Does the law make provision for patients who are incapable of making informed decisions about admission or treatment, but who do not refuse admission or treatment?	No
		2	Are the conditions under which a non-protesting patient may be admitted and treated specified?	No
		3	Does the law state that if users admitted or treated under this provision object to their admission or treatment they must be discharged or treatment stopped unless the criteria for involuntary admission are met?	No
I	Involuntary admission (when separate from treatment) and involuntary treatment (where admission and treatment are combined)	1a	Does the law state that involuntary admission may only be allowed if there is evidence of mental disorder of specified severity?	Yes
		1b	Does the law state that involuntary admission may only be allowed if there is serious likelihood of harm to self or others and/or substantial likelihood of serious deterioration in the patient’s condition if treatment is not given?	Yes
		1c	Does the law state that involuntary admission may only be allowed if admission is for a therapeutic purpose?	No
		2	Does the law state that two accredited mental health care practitioners must certify that the criteria for involuntary admission have been met?	Yes
		3	Does the law insist on accreditation of a facility before it can admit involuntary patients?	Yes
		4	Is the principle of the least restrictive environment applied to involuntary admissions?	Yes
		5	Does the law make provision for an independent authority (e.g. review body or tribunal) to authorise all involuntary admissions?	Yes
		6	Are speedy time frames laid down within which the independent authority must make a decision?	Yes
		7	Does the law insist that patients, families and legal representatives be informed of the reasons for admission and of their rights of appeal?	No
		8	Does the law provide for a right to appeal an involuntary admission?	Yes
		9	Does the law include a provision for time-bound periodic reviews of involuntary (and long-term “voluntary”) admission by an independent authority?	No
		10	Does the law specify that patients must be discharged from involuntary admission as soon as they no longer fulfil the criteria for involuntary admission?	Yes
J	Involuntary treatment (when separate from involuntary admission)	1a	Does the law set out the criteria that must be met for involuntary treatment, including: Patient suffers from a mental disorder?	Yes
		1b	Does the law set out the criteria that must be met for involuntary treatment, including: Patient lacks the capacity to make informed treatment decisions?	Yes
		1c	Does the law set out the criteria that must be met for involuntary treatment, including: Treatment is necessary to bring about an improvement in the patient’s condition, and/or restore the capacity to make treatment decisions, and/or prevent serious deterioration, and/or prevent injury or harm to self or others?	No
		2	Does the law ensure that a treatment plan is proposed by an accredited practitioner with expertise and knowledge to provide the treatment?	Yes
		3	Does the law make provision for a second practitioner to agree on the treatment plan?	No
		4	Has an independent body been set up to authorise involuntary treatment?	Yes
		5	Does the law ensure that treatment is for a limited time period only?	Yes
		6	Does the law provide for a right to appeal involuntary treatment?	Yes
		7	Are there speedy, time-bound, periodic reviews of involuntary treatment in the legislation?	Yes
K	Proxy consent for treatment	1	Does the law provide for a person to consent to treatment on a patient’s behalf if that patient has been found incapable of consenting?	Yes
		2	Is the patient given the right to appeal a treatment decision to which a proxy consent has been given?	No
		3	Does the law provide for use of “advance directives” and, if so, is the term clearly defined?	Yes
L	Involuntary treatment in community settings	1	Does the law provide for involuntary treatment in the community as a “less restrictive” alternative to an inpatient mental health facility?	No
		2	Are all the criteria and safeguards required for involuntary inpatient treatment also included for involuntary community-based treatment?	No
M	Emergency situations	1	Are the criteria for emergency admission/treatment limited to situations where there is a high probability of immediate and imminent danger or harm to self and/or others?	Yes
		2	Is there a clear procedure in the law for admission and treatment in emergency situations?	Yes
		3	Does the law allow any qualified and accredited medical or mental health practitioner to admit and treat emergency cases?	Yes
		4	Does the law specify a time limit for emergency admission (usually no longer than 72 h)?	Yes
		5	Does the law specify the need to initiate procedures for involuntary admission and treatment, if needed, as soon as possible after the emergency situation has ended?	No
		6	Are treatments such as ECT, psychosurgery and sterilization, as well as participation in clinical or experimental trials outlawed for people held as emergency cases?	Yes
		7	Do patients, family members and personal representatives have the right to appeal against emergency admission/treatment?	No
N	Determinations of mental disorder	1a	Does the legislation Define the level of skills required to determine mental disorder?	No
		1b	Does the legislation specify the categories of professionals who may assess a person to determine the existence of a mental disorder?	No
		2	Is the accreditation of practitioners codified in law and does this ensure that accreditation is operated by an independent body?	Yes
O	Special treatments	1	Does the law prohibit sterilization as a treatment for mental disorder?	Yes
		1a	Does the law specify that the mere fact of having a mental disorder should not be a reason for sterilization or abortion without informed consent?	No
		2	Does the law require informed consent for major medical and surgical procedures on persons with a mental disorder?	Yes
		2a	Does the law allow medical and surgical procedures without informed consent, if waiting for informed consent would put the patient’s life at risk?	Yes
		2b	In cases where inability to consent is likely to be long term, does the law allow authorization for medical and surgical procedures from an independent review body or by proxy consent of a guardian?	Yes
		3	Are psychosurgery and other irreversible treatments outlawed on involuntary patients?	No
		3a	Is there an independent body that makes sure there is indeed informed consent for psychosurgery or other irreversible treatments on involuntary patients?	Yes
		4	Does the law specify the need for informed consent when using ECT?	No
		5	Does the law prohibit the use of unmodified ECT?	Yes
		6	Does the law prohibit the use of ECT in minors?	No
P	Seclusion and restraint	1	Does the law state that seclusion and restraint should only be utilized in exceptional cases to prevent immediate or imminent harm to self or others?	Yes
		2	Does the law state that seclusion and restraint should never be used as a means of punishment or for the convenience of staff?	Yes
		3	Does the law specify a restricted maximum time period for which seclusion and restraints can be used?	No
		4	Does the law ensure that one period of seclusion and restraint is not followed immediately by another?	No
		5	Does the law encourage the development of appropriate structural and human resource requirements that minimize the need to use seclusion and restraints in mental health facilities?	No
		6	Does the law lay down adequate procedures for the use of seclusion and restraints, including: who should authorise it; that the facility should be accredited; that the reasons and duration of each incident be recorded in a database and made available to a review board; and that family members/carers and personal representatives be immediately informed when the patient is subject to seclusion and/or restraint?	Yes
Q	Clinical and experimental research	1	Does the law state that informed consent must be obtained for participation in clinical or experimental research from both voluntary and involuntary patients who have the ability to consent?	Yes
		2a	Where a person is unable to give informed consent (and where a decision has been made that research can be conducted): Does the law ensure that proxy consent is obtained from either the legally appointed guardian or family member, or from an independent authority constituted for this purpose?	Yes
		2b	Where a person is unable to give informed consent (and where a decision has been made that research can be conducted): Does the law state that the research cannot be conducted if the same research could be conducted on people capable of consenting, and that the research is necessary to promote the health of the individual and that of the population represented?	Yes
R	Oversight and review mechanisms	1	Does the law set up a judicial or quasi-judicial body to review processes related to involuntary admission or treatment and other restrictions of rights?	Yes
		1a(i)	Does the above body: Assess each involuntary admission/treatment?	No
		1a(ii)	Does the above body entertain appeals against involuntary admission and/or involuntary treatment?	Yes
		1a(iii)	Does the above body review the cases of patients admitted on an involuntary basis (and long-term voluntary patients)?	No
		1a(iv)	Does the above body regularly monitor patients receiving treatment against their will?	Yes
		1a(v)	Does the above body Authorise or prohibit intrusive and irreversible treatments (such as psychosurgery and ECT)?	Yes
		1b	Does the composition of this body include an experienced legal practitioner and an experienced health care practitioner, and a “wise person” reflecting the “community” perspective?	Yes
		1c	Does the law allow for appeal of this body’s decisions to a higher court?	Yes
		2	Does the law set up a regulatory and oversight body to protect the rights of people with mental disorders within and outside mental health facilities?	Yes
		2a(i)	Does the above body conduct regular inspections of mental health facilities?	Yes
		2a(ii)	Does the above body provide guidance on minimising intrusive treatments?	No
		2a(iii)	Does the above body maintain statistics; on, for example, the use of intrusive and irreversible treatments, seclusion and restraints?	No
		2a(iv)	Does the above body maintain registers of accredited facilities and professionals?	Yes
		2a(v)	Does the above body report and make recommendations directly to the appropriate government minister?	Yes
		2a(vi)	Does the above body publish findings on a regular a basis?	No
		2b	Does the composition of the body include professionals (in mental health, legal, social work), representatives of users of mental health facilities, members representing families of people with mental disorders, advocates and lay persons?	Yes
		2c	Is this body’s authority clearly stated in the legislation?	Yes
		3a	Does the legislation outline procedures for submissions, investigations and resolutions of complaints?	Yes
		3b(i)	Does the law stipulate the time period from the occurrence of the incident within which the complaint should be made?	No
		3b(ii)	Does the law stipulate a maximum time period within which the complaint should be responded to, by whom and how?	Yes
		3b(iii)	Does the law stipulate the right of patients to choose and appoint a personal representative and/or legal counsel to represent them in any appeals or complaints procedures?	No
		3b(iv)	Does the law stipulate the right of patients to an interpreter during the proceedings, if necessary?	Yes
		3b(v)	Does the law stipulate the right of patients and their counsel to access copies of their medical records and any other relevant reports and documents during the complaints or appeals procedures?	Yes
		3b(vi)	Does the law stipulate the right of patients and their counsel to attend and participate in complaints and appeals procedures?	Yes
S	Police responsibilities	1	Does the law place restrictions on the activities of the police to ensure that persons with mental disorders are protected against unlawful arrest and detention, and are directed towards the appropriate health care services?	Yes
		2	Does the legislation allow family members, carers or health professionals to obtain police assistance in situations where a patient is highly aggressive or is showing out-of-control behaviour?	Yes
		3	Does the law allow for persons arrested for criminal acts, and in police custody, to be promptly assessed for mental disorder if there is suspicion of mental disorder?	Yes
		4	Does the law make provision for the police to assist in taking a person to a mental health facility who has been involuntarily admitted to the facility?	No
		5	Does the legislation make provision for the police to find an involuntarily committed person who has absconded and return him/her to the mental health facility?	Yes
T	Mentally ill offenders	1	Does the legislation allow for diverting an alleged offender with a mental disorder to the mental health system in lieu of prosecuting him/her, taking into account the gravity of the offence, the person’s psychiatric history, mental health state at the time of the offence, the likelihood of detriment to the person’s health and the community’s interest in prosecution?	Yes
		2	Does the law make adequate provision for people who are not fit to stand trial to be assessed, and for charges to be dropped or stayed while they undergo treatment?	Yes
		2a	Are people undergoing such treatment given the same rights in the law as other involuntarily admitted persons, including the right to judicial review by an independent body?	Yes
		3	Does the law allow for people who are found by the courts to be “not responsible due to mental disability” to be treated in a mental health facility and to be discharged once their mental disorder sufficiently improves?	Yes
		4	Does the law allow, at the sentencing stage, for persons with mental disorders to be given probation or hospital orders, rather than being sentenced to prison?	Yes
		5	Does the law allow for the transfer of a convicted prisoner to a mental health facility if he/she becomes mentally ill while serving a sentence?	Yes
		5a	Does the law prohibit keeping a prisoner in the mental health facility for longer than the sentence, unless involuntary admission procedures are followed?	No
		6	Does the legislation provide for secure mental health facilities for mentally ill offenders?	Yes
U	Discrimination	1	Does the law include provisions aimed at stopping discrimination against people with mental disorders?	Yes
V	Housing	1	Does the law ensure non-discrimination of people with mental disorders in the allocation of housing?	Yes
		2	Does the law make provision for housing of people with mental disorders in state housing schemes or through subsidized housing?	Yes
		3	Does the legislation make provision for housing in halfway homes and long-stay, supported homes for people with mental disorders?	Yes
W	Employment	1	Does the law make provision for the protection of persons with mental disorders from discrimination and exploitation in the work place?	Yes
		2	Does the law provide for “reasonable accommodation” for employees with mental disorders, for example by providing for a degree of flexibility in working hours to enable those employees to seek mental health treatment?	Yes
		3	Does the law provide for equal employment opportunities for people with mental disorders?	No
		4	Does the law make provision for the establishment of vocational rehabilitation programmes and other programmes that provide jobs and employment in the community for people with mental disorders?	Yes
X	Social security	1	Does legislation provide for disability grants and pensions for people with mental disabilities?	No
		2	Does the law provide for disability grants and pensions for people with mental disorders at similar rates as those for people with physical disabilities?	Yes
Y	Civil issues	1	Does the law uphold the rights of people with mental disorders to the full range of civil, political, economic, social and cultural rights to which all people are entitled?	Yes
Z	Protection of vulnerable groups			
	Protection of minors	1	Does the law limit the involuntary placement of minors in mental health facilities to instances where all feasible community alternatives have been tried?	Yes
		2a	If minors are placed in mental health facilities, does the legislation stipulate that they should have a separate living area from adults?	Yes
		2b	If minors are placed in mental health facilities, does the legislation stipulate that the environment is age appropriate and takes into consideration the developmental needs of minors?	Yes
		3	Does the law ensure that all minors have an adult to represent them in all matters affecting them, including consenting to treatment?	Yes
		4	Does the law stipulate the need to take the opinions of minors into consideration on all issues affecting them (including consent to treatment), depending on their age and maturity?	No
		5	Does legislation ban all irreversible treatments for children?	No
	Protection of women	1	Does legislation allow women with mental disorders equal rights with men in all matters relating to civil, political, economic, social and cultural rights?	Yes
		2a	Does the law ensure that women in mental health facilities: have adequate privacy?	No
		2b	Does the law ensure that women in mental health facilities: are provided with separate sleeping facilities from men?	No
		3	Does legislation state that women with mental disorders should receive equal mental health treatment and care as men, including access to mental health services and care in the community, and in relation to voluntary and involuntary admission and treatment?	Yes
	Protection of minorities	1	Does legislation specifically state that persons with mental disorders should not be discriminated against on the grounds of race, colour, language, religion, political or other opinions, national, ethnic or social origin, legal or social status?	Yes
		2	Does the legislation provide for a review body to monitor involuntary admission and treatment of minorities and ensure non-discrimination on all matters?	No
		3	Does the law stipulate that refugees and asylum seekers are entitled to the same mental health treatment as other citizens of the host country?	No
AZ	Offences and penalties	1	Does the law have a section dealing with offences and appropriate penalties?	Yes
		2	Does the law provide appropriate sanctions against individuals who violate any of the rights of patients as established in the law?	Yes

This table comprises a slightly edited version of the World Health Organization’s “Checklist on mental health legislation” (WHO (2005) WHO resource book on mental health, human rights and legislation. Geneva: WHO)

The table indicates whether legislation in India meets or does not meet specific standards

See text for details and references in relation to individual standards

Overall, then, India’s compliance with the WHO-RB standards is generally good. It is more concordant with the WHO-RB than the legislation of Ireland or England and Wales [33]. There is, however, a number of areas of low compliance. Some areas show just significant *semantic* differences between the WHO-RB and the IMHA, which may stem in part from the IMHA’s attempt to align with the UN-CRPD. These differences reflect the somewhat different theoretical underpinnings of the two documents and have at times complicated the comparison. These areas are discussed below, but first it is useful to note areas of good concordance with the WHO-RB that attributable to legislation other than the IMHA.

### Areas of good concordance with the WHO-RB outside the IMHA

Areas of good concordance in Indian legislation outside of the IMHA are summarised in Table 2. The RPDA does much in the area of employment but it may not be sufficient. It protects four per cent of Government jobs for individuals with benchmark disabilities: one per cent is for individuals with visual impairment, one per cent for individuals with hearing impairment and one per cent for individuals with locomotor disability. This leaves one per cent divided between autism, intellectual disability, specific learning disability, mental illness, and multiple disabilities.^1^ In light of the prevalence of mental illness (above) [3, 7], this does not provide for equal employment opportunities for individuals with mental illness as required by the WHO-RB (W3).

**Table 2:** Areas where Indian legislation outside of the Indian Mental Healthcare Act (IMHA) is concordant with the World Health Organization’s “Checklist on Mental Health Legislation” (WHO-RB) (World Health Organization, 2005)

Legislative issue	WHO-RB designation	Relevant Indian legislation outside the IMHA
Discrimination	U	Rights of persons with disabilities Act (RPDA) 2016, sections 2(h) & 3
Housing	V	RPDA 2016, sections 12(3), 18(4)(b), 19(3), 65(2)(e), 71(2)(e) & 121(4)(b)
Employment	W	RPDA 2016, sections 19, 20 & 35
Social security	X	RPDA 2016, section 24
Civil issues	Y	RPDA 2016, sections 12(1), 16(i) & 20

### Areas of low concordance with the WHO-RB across all Indian legislation

Key areas of low concordance with the WHO-RB across all Indian legislation are summarised in Table 3. Families’ rights in the day-to-day treatment of their relatives are, essentially, accorded to the individual’s nominated representative. As the individual receiving mental healthcare may revoke an appointment of a nominated representative at any time,^2^ the individual’s family or carers have no protected right to information (E1), treatment planning (E2) or appeal (E3). There is no provision for anyone to apply for the discharge of mentally ill offenders (E4). Two occasions exist when the family and carers are automatically involved; these are: when planning discharge^3^ and in the case of a person found wandering in the community.^4^

**Table 3:** Areas of low concordance between the Indian Mental Healthcare Act (IMHA) and the World Health Organization’s “Checklist on Mental Health Legislation” (WHO-RB) (World Health Organization, 2005)

Legislative issue	WHO-RB designation	Area of omission
The rights of the family	E1-4	Limited rights when they are not the nominated representative
Non-protesting patients	H1-3	Not considered in the IMHA, patients lacking capacity admitted as a supported admission
Involuntary treatment in the community	L1-2	Not directly mentioned outside the context of emergencies, no clear safe guards
Determination of mental illness	N1a-b	No guidelines for necessary level of training or category of professions who can determine mental illness
The rights of minorities	Z2-3	No review body monitors the involuntary admission of minorities and no reference is made to refugees or asylum seekers

Involuntary treatment in the community setting (L) is only referred to in the context of emergencies.^5^ The legislation concerning advance directives and capacity does create the possibility for involuntary community treatment (L1) (below) but lacks clear criteria and safeguards (L2).

### Areas where comparison is complex

Section I of the WHO-RB deals with involuntary admission and treatment, and comparison with the IMHA is in some ways limited as the IMHA does not legislate for involuntary admission directly. A person may, however, be admitted against their will using “supported admission”,^6^ so we assessed this procedure in relation to the WHO guidelines. Supported admissions are only allowed if there is evidence of mental disorder of a specified severity (I1a)^7^; if the individual is posing a risk to them self or others or is unable to care for themselves (I1b)^8^; and if two accredited mental health professionals agree that the individual meets the given criteria (I2).^9^ The admission must be to a registered mental health establishment (I3)^10^ and must be the least restrictive care option (I4).^11^

Under the IMHA, the relevant Mental Health Review Board (MHRB) is informed within seven days of a supported admission (three days in the case of a minor or woman).^12^ The admitted person, their nominated representative or an appropriate organisation may appeal this decision (I8).^13^ No automatic review process occurs during the initial admission order. If a section 89 admission continues for its maximum thirty days and ongoing supported admission is required, this can continue under section 90. At this stage, the MHRB is informed and they must review the admission (I5)^14^ within twenty-one days (I6),^15^ and either permit the admission or order discharge of the individual. These reviews of a supported admission continue at a maximum frequency of 180 days (I9).^16^ Should an individual no longer fulfil criteria for a supported admission the supported admission must be terminated (I10).^17^

As highlighted in our consideration of Section E, families are not always entitled to information concerning the admitted individual. The WHO-RB suggests that patients, family and legal representatives be informed of the reasons for admission and of their rights of appeal (I7). In India, this information is to be imparted through the nominated representative, who, if not already selected by an advance directive, is ideally a relative or career. This may leave family members in a situation where they are not entitled to any information or to appeal an admission. The Indian Act does, however, direct the medical officer to provide the individual and their nominated representative with information concerning the admission, the IMHA and their right to appeal.^18^

The Indian Act does not include serious likelihood of deterioration as a criterion for admission, as suggested by the WHO-RB (I1b). There is also no mention of the need for the admission to be for therapeutic purposes (I1c); this is, however, a requirement for independent admissions^19^ and is alluded to in the IMHA as supported admission has to be considered to be the least restrictive care option.^20^ No periodic reviews occur for long-term voluntary adult patients (I9), although mandatory review does occur in the case of minors who are admitted (after thirty days) (I9).^21^

Comparison between the IMHA and the WHO-RB is difficult in the area of involuntary treatment (when separate from involuntary admission) (J). The IMHA states that all persons have the capacity to make treatment decisions but may require varying levels of support from their nominated representative^22^; consequently, treatments are not “involuntary”. The IMHA proposes creating a guidance document to aid medical practitioners in assessing an individual’s capacity to make treatment decisions,^23^ with independent patients defined as having the capacity to make such decisions.^24^

Under the IMHA, treatment without informed consent can only be given in the context of a supported admission; for this, the patient must be suffering from a mental disorder (J1a)^25^ and lack the capacity to make informed treatment decisions (J1b).^26^ The IMHA ensures that a sufficiently qualified practitioner provides a treatment plan (J2) by laying out defining criteria for relevant mental health specialties^27^ and ensuring that patients receive a treatment plan as soon as possible after admission.^28^ The duration (J5), review (J4, J7) and appeal (J6) processes for supported treatments are as for a supported admission.

While posing a risk to oneself or others or an inability to care for oneself are considered reasons for supported admission,^29^ no reference is made to the need to improve a patient’s condition, restore decision making capacity or prevent deterioration (J1c). No second opinion is needed for a supported treatment plan (J3), only for admission.

Regarding proxy consent for treatment, the IMHA and the WHO-RB are relatively well aligned. The IMHA provides for a nominated representative to consent on a patient’s behalf (K1) if the patient requires nearly one hundred per cent support from them.^30^ The IMHA also provides for and clearly defines advance directives (K3).^31^ While there is no clear mechanism to appeal against a treatment decision to which proxy consent has been given (K2), the individual’s capacity is reviewed every seven to fourteen days^32^ and the individual has the right to revoke the appointment of a nominated representative at any time.^33^

### Key omissions in areas of generally good concordance

Many of the twenty-seven sections of the WHO-RB are generally well covered in Indian legislation but, commonly, one or two key standards are not met. These omissions are summarized in Table 4 and discussed in more detail below.

**Table 4:** Key omissions in Indian legislation in areas of otherwise good concordance with the World Health Organization’s “Checklist on Mental Health Legislation” (WHO-RB) (World Health Organization, 2005)

Legislative issue	WHO-RB designation	Key omissions
Preamble and objectives	A2a	Failure to emphasise non-discrimination outside of healthcare
Definitions	B3	No discussion of personality disorder No clear definitions concerning substance misuse
Access to mental healthcare	C1	No clear plans for funding services
Rights of users of mental health services	D5 D6a	No minimum conditions for mental health establishments Superficial consideration of privacy
Competence, capacity and guardianship	F4-7	Lack of clarity and review process concerning limited guardians No clear duration or systematic review for nominated representatives
Emergency situations	M4 M7	No concept of emergency admission No guidelines for appealing against emergency treatments
Special treatments	O1a O2	No explicit protection from abortion without informed consent Lack of guidance on medical treatments
Seclusion and restraint	P3-4 P5	Potential for prolonged and repeated seclusion. No human resource strategy to minimize use of seclusion and restraint
Oversight and review mechanisms	R1ai R1aiii R1aiv R2aiii-iv	Not all involuntary admissions are automatically reviewed Long-term voluntary patients have no right to external review No review for treatments against an individuals will No guidelines on the collection and publication of statistics
Protection of vulnerable groups	Z2a Z2b	The privacy of women is not given special consideration Women are not guaranteed separate sleeping facilities from men

No reference is made in the IMHA to personality disorder (B3) which could be considered a mental illness according to the definition included. The legislation is also ambiguous concerning substance abuse (B3), referring to “mental conditions associated with the abuse of alcohol and drugs”.^34^ This could include intoxication, harmful use of substances, substance dependence, withdrawal, drug induced psychosis and brain damage secondary to substance misuse. The precise extent of this definition requires clarification.

Moreover, it is not, as the WHO-RB requires, clear why particular terms have been chosen (B2), especially as the IMHA aligns itself with the International Classification of Disease,^35^ but does not use its terminology.

Competency, capacity and guardianship (F) is one of the most important areas where the Indian legislation has low levels of concordance with the WHO-RB, addressing only 50% (4/8) of relevant WHO-RB items. Despite this, the IMHA comprehensively covers a number of important issues. Regarding provision for managing the affairs of people with mental disorders (if they are unable to do so themselves) (F1), the IMHA lays out guidelines for advance directives^36^ and nominated representatives who would be able to address individual’s healthcare decisions should the need arise.^37^ Other matters could be addressed through guardianship (below). “Capacity” is clearly defined^38^ and while “competency” is not (F2), this is nonetheless sufficient to meet the relevant WHO-RB standard (F2). The IMHA also lays down criteria for determining capacity^39^ and proposes the development of a guidance document for assessing it.^40^

In the IMHA, capacity, advance directives and nominated representatives only pertain to decisions concerning healthcare; no reference is made to financial decisions. Guardianship, which may cover financial and other decisions, is addressed in the RPDA^41^ and runs in parallel with the nominated representative in the IMHA. The RPDA describes the provision of a limited guardian who may take legally binding decisions for “a person with disability who has been provided with adequate and appropriate support but is unable to take legally binding decisions”.^42^ The procedure for the appointment of a limited guardian is clearly described, but the duties, duration (F5) and areas of responsibilities (F6) of a limited guardian are to be determined by the State governments. In the RPDA, there is no provision for systematic reviews (F7), though this may be prescribed by State governments, and there is the right to appeal the decision (F8).^43^

The IMHA is very clear on the appointment,^44^ duties and responsibilities^45^ of nominated representatives, though it should be noted that these people do not act on behalf of the individual in the manner described in the WHO-RB (F5), but rather support the decision-making of the individual. The IMHA, however, offers no guidelines about the duration of the nominated representative’s activities and this may have important consequences in the future (below).

Emergency situations are addressed in a similar manner in both the IMHA and WHO-RB (M). Emergency treatments are time limited to 72 h (M4)^46^ except in North-East and Hill States where it is extended to 120 h due to local infrastructure.^47^ While research is not explicitly forbidden in emergency situations, it would require ethical approval and would have to comply with all national and international guidelines.^48^

The IMHA does not state that there is a need to initiate procedures for supported admission and treatment, if needed, as soon as possible after the emergency situation has ended (M5). However, transportation of a person to a mental health establishment is part of emergency treatment.^49^ If the nominated representative is present, emergency treatment may only occur with their consent.

No mention is made of abortion in the IMHA. Terminations of pregnancy are permitted in certain contexts^50^ with the woman’s consent^51^ under the Medical Termination of Pregnancy Act, 1971. Provision is made for women lacking capacity^52^ and this is clarified in the RPDA which states that it may be allowed in cases of severe disability where the opinion of the medical practitioner and the guardian of the woman with disability are considered.^53^ This does not, however, give explicit protection from abortion without informed consent to individuals with mental illness as required by the WHO-RB (O1a).

The IMHA makes only one reference to medical treatments and none to surgical treatments (except psychosurgery) (O2). In an emergency, “any medical treatment” can be given if it directly relates to the emergency.^54^ While this may cover an episode of delirium, it cannot apply inside of a mental health establishment or to a supported patient. No direct reference is made to the need for informed consent prior to medical and surgical procedures. However, the government is to ensure that persons with disabilities enjoy legal capacity on an equal basis with others (O2)^55^ and, on this basis, consent would be required from all individuals capable of giving it. Where that capacity to give informed consent is absent either in an emergency situation (O2a) or in the long term (O2b), a limited guardian may make legally binding decisions on behalf of the individual without capacity.^56^

To deal with oversight and review mechanisms required by the WHO-RB (R), three bodies are empowered in the IMHA: the Mental Health Review Boards (MHRB), the State Mental Health Authorities (SMHA) and the Central Mental Health Authority (CMHA).

The MHRBs are responsible for reviewing supported admissions, advance directives, nominated representatives and adjudicating complaints (R1)^57^ and appeals (R1aii).^58^ MHRBs are informed of all supported admissions but within the first thirty days they only review them at the request of the person admitted or their nominated representative.^59^ Should supported admission be required after the initial 30 days a section 90 admission can be started and following this the MHRB will review the admission.^60^ Should an individual feel aggrieved by the decision of a MHRB, they may appeal to the High Court of the State (R1c).^61^ The MHRBs comprise a district judge, a representative of the district, a psychiatrist, a second medical practitioner and two individuals who suffer from mental illness or are care-givers or representatives of organisations advocating for those with mental illness (R1b).^62^

The IMHA sets up the SMHA and CMHA which will provide regulation and oversight in many areas of mental health (R2). The authority of the CMHA^63^ and SMHA^64^ are not clearly laid out (R2c) but their roles are covered to some extent in the IMHA. They will conduct inspections of mental health establishments (R2ai),^65^ and maintain a list of registered clinical psychologists, mental health nurses and psychiatric social workers (R2aiv). Guidelines concerning psychiatrists are covered under the Indian Medical Council Act, 1956.^66^ No reference is made to occupational therapists, counsellors, psychotherapists or other specialties working within mental health.

Both the SMHA and CMHA include representatives of the Department of Health and Family Welfare and advise their respective governments (R2av).^67^ The SMHA^68^ and the CMHA^69^ are composed of a wide range of mental health professionals, service users and their representatives, as required by the WHO-RB (R2b).

The MHRBs and the Central and State Authorities will engage in activities which may reduce intrusive treatments but will not produce guidelines or take comprehensive steps to minimize such treatments (R2aii). They plan to develop quality and service provision norms^70^ and prepare a guidance document on assessing capacity,^71^ both of which may limit the use of intrusive treatments. The MHRB in consultation with the SMHA can take measures to protect the rights of persons with mental illness.^72^ The CMHA may also give direction to further regulate the use of seclusion, restraint and psychosurgery. The RPDA also sets up two bodies which may reduce intrusive treatments, these are the State^73^ and Central^74^ Advisory Boards on Disability. Their roles include advising government, developing policy, monitoring the impact of laws and taking up the cause of persons with disability.

The IMHA lays out clear procedures for the submission, investigation and resolution of complaints (R3a).^75^ In appeals, an individual has a right to choose their nominated representative^76^ but no mention is made of their legal counsel (R3biii), although all persons with mental illness are entitled to receive free legal services in matters relating to the IMHA.^77^

Regarding the protection of vulnerable groups (Z), minors are well protected but the guidelines are not as comprehensive for women and ethnic minorities. While steps are taken to consider the opinion of the minor (Z4), these only relate to admission^78^ or are dependent on the nominated representative.^79^

With regard to women, the IMHA again only addresses gender-based discrimination in relation to healthcare, and not more broadly (Z4).^80^ The RPDA, however, affirms the equality of men and women in its preamble and legislates that government must take measures to ensure that women attain equal rights with others in all areas of life (Z1).^81^

### Areas of good concordance

The IMHA and the WHO-RB are closely aligned in relation to voluntary admission and treatment (G), guidelines on clinical and experimental research (Q),^82^ police responsibility (S), provisions concerning mentally ill offenders and offences and penalties.

In relation to police responsibility, it does not explicitly state that family members, carers or health professionals can obtain police assistance in situations where a patient is highly aggressive (S2); this is information that the police can consider as a reason to believe that a person has a mental illness.^83^ No special provision is made to allow police to assist in taking a person to a mental health establishment who requires a supported admission (S4), but the return of a person under a supported admission order who absconded from the mental health establishment is addressed (S5).^84^ While the IMHA is clear on the role and responsibilities of police regarding individuals in the community and prisoners serving a custodial sentence, it is less clear concerning persons arrested for criminal acts (S3). It does give provision for a magistrate to convey such a person to a mental health establishment if required.^85^

The provisions concerning mentally ill offenders (T) are limited in the IMHA but relevant matters are covered in the Code of Criminal Procedure and the Indian Penal Code. The IMHA allows for the transfer of a convicted prisoner to a mental health facility if required (T5)^86^ but the law does not prohibit the keeping of a prisoner in the mental health facility for longer than the sentence (T5a). To provide forensic mental health facilities, the IMHA legislates for mental health establishments (T6) to be created in the medical wing of at least one prison in each State.^87^ Even though no provision is made for secure forensic psychiatry services outside of the prisons, this is still significant progress.

Two sections of the IMHA apply to individuals at the sentencing stage (T3, T4) and these create a provision where an individual may be brought to hospital.^88^ The law states that nothing is an offense if, by reason of unsound mind, the person could not know the nature or illegality of an act (T4).^89^ The appropriate diversion of offenders with mental health disorders *in lieu* of prosecution is briefly alluded to in the IHMA^90^ but it does not give special consideration to the gravity of the offence, the person’s psychiatric history, mental health state at the time of the offence, the likelihood of detriment to the person’s health, or the community’s interest in prosecution, as required by the WHO-RB (T1). The IMHA also allows some provision for people who are not fit to stand trial (T2); this is covered in more depth in the Code of Criminal Procedure^91^ and, if transferred to a mental health establishment, such persons are given the same rights and protections that all patients receive under the IMHA (T2a).^92^

### Areas of well justified non-concordance

Psychosurgery is not forbidden in involuntary patients as suggested by the WHO-RB (O3) but it is only permitted with the consent of the individual and with approval of a MHRB (O3a). Regarding ECT, unmodified procedures are prohibited (O5).^93^ Informed consent is, however, not required (O4), although consent can be obtained from the nominated representative.^94^ With the permission of a guardian and the MHRB, ECT can be delivered to a minor (O6).^95^ As discussed below, these departures from the WHO-RB may actually serve to enhance the rights of individuals with mental illness.

## Discussion

The discussion section of this paper follows the order of the Results section and expands on complex or contentious issues. Areas of good compliance have been mostly omitted from further consideration so as to focus on areas in need of further attention and improvement.

### Areas of good concordance with the WHO-RB outside the IMHA

The RPDA is the main piece of legislation that fulfils the WHO-RB outside of the IMHB. The IMHB and the RPDA have different roles. The IMHB applies to only mental illness, while the RPDA includes all individuals with disabilities. As such the IMHA addresses emergency situations not considered in the RPDA. This focus on acute episodes by the IMHB, in contrast to the RPDA can also be seen in relation to proxy decision making. The role of the limited guardian in the RPDA is more holistic and long-term, compared to that of the nominated representative in the IMHA. Both documents are needed to address the varying components of the WHO-RB and while they usually do this in a complementary manner, there are potential areas of conflict between the two acts. This is particularly relevant in the areas of proxy decision making, capacity and supported admission and treatment.

The right to equality and non-discrimination is affirmed in the IMHA,^96^ although this only relates to healthcare and not to broader discrimination in other areas of life owing to mental illness. While some of these other areas are addressed in the RPDA, this is still a stark omission from national mental health legislation. This is especially true in light of the role of stigma in preventing people accessing treatment [34] and the prevalence of stigma in mental health [35]. In addition, it is known that stigma in India can have far-reaching consequences outside of access to healthcare [36]. Hence, more specific anti-discrimination provisions are needed in Indian mental health legislation.

A large discrepancy between the IMHA and the WHO-RB concerns mentally ill offenders, but many of these important items are addressed elsewhere in Indian legislation. Forensic psychiatry in India is in development [37] and Kallivayalil et al. [23] astutely recount how the current, unsatisfactory situation has evolved from a long history of governmental apathy. In addition, in order to improve overall coherence, it is clearly important that terminology is consistent across all Indian legislation, as many older acts need updating to remove terms like “lunatic” and affirm a consistent legal stance on capacity which is compatible with the UN-CRPD.

### Areas of low concordance with the WHO-RB across all Indian legislation

We adopted a literal but pragmatic approach to our analysis, and so classified the IMHA as non-concordant with 80% of the rights of family and other carers (E). This group’s rights are, at best, indirect in the IMHA, being facilitated through the role of the nominated representative. Outside of this context, the entitlements of family and carers are extremely limited [38]. Asoken [37] has already raised concerns about how a nominated representative may do more harm than good, and questioned their relevance to Indian culture.

The IMHA’s lack of consideration of non-protesting patients is a particular concern. In 2011, 25% of Indian mental health patients had been in hospital for over 6 months [7]. The rising prevalence of dementia [5] highlights the particular urgency of the need for legislation for this group, as individuals with dementia often require significant support to exercise their capacity.

A robust process for reviewing admissions is also vital. The concerns raised above about non-protesting patients may be addressed by the transition from “involuntary” to “supported” admission. In many jurisdictions non-protesting patients are admitted but without the legal protection afforded by involuntary treatment (i.e. automatic review of their admissions and treatment) [33]. Under the IMHA, if an individual’s capacity is impaired to the extent that they need a high level of support in decision-making, they should then be admitted in a supported manner. This will afford them the necessary protections. We are yet to see how this will be implemented; it is possible that this will be underutilized and many individuals may remain in hospital without their informed consent.

The IMHA does not make specific mention of refugees or asylum seekers. However, at the end of 2015 India had over 200,000 “persons of concern” to the UN High Commissioner for Refugees [39]. These individuals have a high prevalence of mental illness and face many barriers to accessing services; as such, they warrant particular mention and legislative protection [40].

There is also a lack of emphasis on primary health care in the IMHA. This is regrettable because the need for more primary health care has long been recognised in India [41] and services are still insufficient in many areas.

Finally, the WHO-RB suggests that supported admissions should only be allowed if admission is for a therapeutic purpose. Explicitly stating this would prevent supported admissions of individuals who do not benefit from in-patient treatment or are being admitted for primarily social reasons. Again, Indian legislation, like legislation in many other countries, could do significantly more in this regard.

### Areas where comparison is complex

Five specific legal constructs in the new Indian legislation adopt perspectives significantly different to those of the WHO-RB and an understanding of these is vital for any comparison of the two documents. These are: advance directives, supported admissions, nominated representatives, limited guardians, and capacity.

The potential effects of advance directives are vast and as the IMHA currently stands it is unclear how these will be utilized. It is not explicitly stated that advance directives only apply to admission. As such, it is possible that, when an individual ceases to have capacity,^97^ their advance directive may state a preference for supported (involuntary) treatment in the community. This opens up the possibility of supported treatment separate from admission, and possibly a version of community treatment orders with relatively poorly delineated parameters and poor review mechanisms. Asokan [37] might well prove prescient in describing the proposed advance directive legislation as a “Pandora’s box”. Some psychiatrists believe the legislation will limit their ability to treat individuals by giving too much freedom [42]. The Committee on the Rights of Persons with Disabilities, however, appears to take the contrasting view that such provisions are overly restrictive for patients [43]. In addition to these concerns, the advance directive process will likely place additional financial burden on Indian mental healthcare services [42]. Some have suggested that advance directives may result in criminalisation of individuals with mental illness who decline treatment but come into contact with the criminal justice system [44]. The role of advance directives in other jurisdictions has been called into question [45] and requires further careful, constructive thought.

A second construct which hinders direct comparison between the WHO-RB and the IMHA is that of supported admission. The UN-CRPD appears opposed to involuntary treatment [43]. To adhere with this, the individual’s capacity is supported by their nominated representative under the IMHA. As a result, the individual with mental illness apparently never entirely loses capacity to make decisions; instead, capacity requires varying degrees of support. While there is an ambiguity in this process, it is an arguably necessary ambiguity owing to the complex and varied situations in which this legislation will apply.

Essentially, what would have been considered an involuntary admission in the past is now to be an admission where an individual requires “a very high level of support, approaching hundred percent support in making decisions”.^98^ This is a departure from the WHO-RB which suggests the appointment of a guardian who can make decisions in place of the individual.

This provision of the IMHA is undoubtedly an admirable effort to maximise the rights and autonomy of individuals with mental illness, but the non-dichotomous classification of supported decision-making may reduce an individual’s access to the review process. In other words, the IMHA’s approach protects the individual’s capacity but at a cost. The role of the nominated representative is not subject to sufficient systematic review and lacks discrete time frames; and it is unclear what form that support takes or to what degree it is binding. This opens up the potential for loss of autonomy without the protection of a review process. It seems to be an idealistic abdication of responsibility to move the limitation of rights from trained professionals, acting in accordance with professional standards, to family members or others, with potentially varied priorities and limited experience.

It is unclear to what extent supported admissions will be used. They may be used in the cases of non-protesting patients in addition to patients refusing treatment. While the potential delay (up to 51 days) prior to automatic review is a source of concern, it is also important that timeframes are realistic in the context of services’ ability to deliver reviews on time. The WHO highlighted the current limitations in the workforce in Indian mental healthcare noting that there are just 0.30 psychiatrists, 0.17 nurses, 0.05 psychologists and 0.03 social workers per 100,000 population [8]. Figures for all mental health professional are increasing, though many of these individuals are working in the private sector and exact numbers have not been obtained from many states [7]. Ultimately, issues such as this may well prove the rate-limiting step in implementing the IMHA.

Notwithstanding these concerns, the construct of nominated representative can still be used to fulfil many of the requirements of the WHO-RB. Legally and pragmatically, this tool has strengths and weaknesses. According to the IMHA, *every* adult has the right to appoint a nominated representative.^99^ The nominated representative may be any adult who is competent to perform the “duties assigned to him” and has given written consent to acting in this capacity.^100^ If a person has not appointed a nominated representative, one can be appointed for them. The nominated representative advocates for the individual and supports their capacity and decision-making. As already discussed (above), this arrangement avoids treatment being seen as “involuntary”. Consistent with this, an individual may “revoke or alter such appointment at any time”.^101^ The role of nominated representative may also, however, limit the role of carers and family members in certain situations [38].

There is a complex and ill-defined relationship between the role of the IMHA-nominated representative and that of the limited guardian under the RPDA. The former concept is more in-line with the UN-CRPD Committee on the Rights of Persons with Disabilities and harmonisation with the UN-CRPD is clearly important for India (and other countries) from a legislative perspective [43]. Despite this, the use of a nominated representative clearly has the potential to result in a greater limitation of rights for certain patients in certain circumstances. This is because under the IMHA decision-making capacity is not a binary concept but rather relates to varying levels of support that are required. Therefore, the concept of a person’s capacity being temporally limited for a defined period of time with a clear review process has been replaced with a more nebulous concept where their decision-making is supported by an individual, to an unspecified degree, for a poorly defined duration, with insufficient review.

The absence of a clear duration during which the nominated representative acts may lead to the individual with mental healthcare needs being in coercive situations with very limited review of the “support” they are receiving. While the individual in question has the right to revoke the appointed nominated representative at any time,^102^ there is no clear guidelines that a person needs to have capacity to alter or revoke their appointment. A MHRB may also revoke an appointment if they feel it is in the interests of the individual.^103^ In addition, while some psychiatrists perceive MHRBs as progressive, they have also been met with some skepticism [44]. Concerns have been raised about recruiting sufficient staff for MHRBs, training of individuals on the MHRBs, and potential disagreements between these boards and treating psychiatrists [46].

Potential tensions stemming from the existence of guardianship together with advance directives and nominated representatives all at the same time have yet to be resolved but could raise serious issues [17].

Finally, the concept of capacity itself is a particularly complex one in the IMHA. One issue that arises in the legislation is whether or not capacity is binary and there is no clear answer to this. In relation to advance directives, capacity is dealt with as either present or absent,^104^ but in relation to nominated representatives^105^ and admission,^106^ there is a supportive model where the level of support is adjusted according to the level of capacity. The IMHA proposes creating guidelines for this area but, in the absence of these, the role and assessment of capacity remain unclear.

### Key omissions in areas of generally good concordance

Clear definitions are an essential part of any piece of legislation and as many concepts in psychiatry have a significant subjective component they are particularly important in this field. The UN-CRPD has been hesitant to lay down precise definitions of mental illness and has justified this position as accommodating the evolving understanding of disability and minimising the exclusion of individuals who fall outside of rigid definitions [47]. This position has its drawbacks, however, and may not be tenable for national mental health legislations. For example, it hinders the collection of data which are needed for evidence-based service development.

In addition, as the IMHA sets out to protect the rights of individuals with mental illness, it is important that it can accurately identify such individuals [48]. A clear definition of mental disorder is provided in the Act,^107^ but the failure to identify clearly the position of addiction and personality disorder has important implications. The omission of consideration of personality disorder from the IMHA is particularly problematic.

Under the IMHA, a mental health service has a “duty to provide care, treatment and rehabilitation to a person, having severe stress and who attempted to commit suicide”.^108^ High suicide risk was identified in 0.9% individuals in India so this requirement could put an unmanageable burden on mental healthcare services [3]. Certain personality disorders are associated with high rates of suicidality [49] and individuals with personality disorders can make up a significant proportion of the work-load of psychiatric services. In an American population, approximately 15% of adults have at least one personality disorder [50]. Newton-Howes et al. highlight that up to 50% of individuals attending secondary care have a personality disorder [51]. While rates of personality disorder in India are unclear, it is still the case that if government has a duty to provide care and treatment to anyone who attempts suicide,^109^ it is inevitable that many individuals with personality disorder will come into contact with the mental healthcare services. Failure to make direct reference to personality disorder may lead to individuals with very severe levels of disability not receiving support that they need [17] or it may lead to inappropriate supported admissions and treatment. Greater clarity is needed.

Adequate diagnosis of mental illness is essential for high quality mental healthcare, and the IMHA is regrettably silent on the categories of professionals or skills required to diagnose mental illness. While this may be a product of the work-force limitations highlighted above, it is still essential that some safeguards are in place, as there is a need for a high level clinical training and judgement for accurate diagnosis [50].

No provision is made for financing mental health services in the IMHA, this omission may limit the realisation of the act. The IMHA enshrines the provision of mental healthcare in legislation, a first for India.^110^ The WHO have recently identified the potential for legislation to further healthcare aims [52] and psychiatry is well placed to lead the way in this area [53]. The legal onus to provide healthcare, without clear financial resources, however, runs the risk of being perceived as overly idealistic and impractical. This may result in it not being realized or mental health care being provided at the expense of different sectors.

Privacy is a fundamental human right [15, 29]. It is so important that it is discussed under the section of the IMHA devoted to protection from cruel, inhumane and degrading treatment.^111^ As the protection from such treatment is a non-derogable human right [54], privacy must be protected at all times. Regrettably, discussion concerning privacy is sparse in the medical literature and in much legislation, including the IMHA and UN-CRPD [38]. The WHO-RB, by contrast, discusses varying forms of privacy in considerable detail, and how they might apply in different contexts. Deeper consideration of privacy in the IMHA would do much to protect the rights of individuals receiving mental healthcare in India and would set an important example for other countries.

The human resource limitations in Indian mental health services [7] increase the need for careful regulation of seclusion and restraint. The IMHA’s failure to comply with certain WHO-RB items relating to seclusion and restraint (above and Table 1) may increase the possibility of long durations of seclusion and restraint. With growing movement towards seeing seclusion and restraint as forms of cruel, inhuman or degrading treatment [55], it is essential that these practices, if utilised, are kept within very clearly defined parameters and accompanied by all necessary protections. The large variations in the frequency and duration of seclusion and restraint further underline the need for well-constructed guidelines [56].

A recent study of mental illness in India noted that there are no national level prevalence studies of mental illness in the country [4] although some steps have been taken to address this [3]. The IMHA presented an opportunity to address this data deficit but the legislation does not require even rudimentary data collection. This is regrettable: the limited epidemiological knowledge available impacts significantly on the ability of health service planners and public health officials to address the needs of the mentally ill [5]; national, statutory data collection, underpinned by legislation, would have done much to remedy this deficit.

### Areas of well justified non-concordance

The IMHA makes a small but important, progressive deviation from the WHO-RB in the area of psychosurgery. The WHO-RB suggests that psychosurgery should not be permitted for involuntary patients. This could potentially prevent a supported patient receiving a beneficial treatment. The IMHA correctly identifies decision-making capacity rather than status of admission as the key issue here. This is important: Mandarelli et al. demonstrated high levels of decision-making capacity in patients receiving non-consensual psychiatric treatment [57]. The IMHA affirms that supported patients may retain the capacity to make treatment decisions.

### Weaknesses of this study

The WHO-RB recommends that a committee, from a range of disciplines, undertake analysis of legislation using the WHO-RB [58]. This research was, in contrast, conducted by two psychiatrists; our aim, however, was to use the WHO-RB framework to provide an overview of key human rights issues in the legislation and to stimulate further, broader, multi-disciplinary consideration of these matters. In addition, this analysis was conducted by individuals working outside the Indian system with the explicit aim of optimising analytic objectivity and engaging in a purely black letter analysis; i.e. determining to what extent the IMHA *as written* appears to comply with the WHO-RB *in theory*. This is a necessary first step and, now that this is done, there is a need to move forward and complement this work with further collaborative analysis based on first-hand experience of Indian mental health services focussing on the legislation *in practice.* These two kinds of analysis (in theory and in practice) can produce quite different assessments and both serve important purposes.

This type of research does not consider implementation. It focuses instead on the *content* of legal documents. Implementation is a key issue for mental health legislation internationally and has posed particular challenges in low and middle income countries [59]. Twenty-eight years after the enactment of the Mental Health Act 1987, only eleven percent of Indian states had state mental health rules in place and it is suggested that many states were unaware of these rules [7]. Specific measures need to be taken to address common barriers to full implementation which include (but are not limited to) funding, staffing, public health priorities and stigma [60]. Concerns regarding implementation justifiably cast a shadow on the new legislation and warrant future research.

## Conclusions

The IMHA is a significant step towards greater recognition and protection of the rights of the mentally ill in India. Such a comprehensive attempt to align national mental health legislation with the UN-CRPD is not only admirable in itself but will surely influence many other countries to do likewise; this is greatly to be welcomed. It is imperative, however, that the Indian legislation does not follow the same path as the Indian asylums described by Wig [11]. These asylums opened with “great hope and expectation” but within a few years become overwhelmed. Lack of services, lack of investment, and demoralisation among service providers resulted in serves-users being neglected despite the original enthusiasm and investment. Failure to invest financially or politically in the realisation of this new Act on the ground could see the IMHA similarly fail the people it seeks to protect. A good piece of legislation that is poorly implemented might well be more damaging to patients than a poor piece of legislation that is implemented well.

There are also specific matters in the IMHA still in need of remedy. References to discrimination on the basis of mental illness relate only to healthcare, and while the RPDA partially addresses this, mental illness still requires further and special consideration in this regard. Specific legislation concerning mental illness would better safeguard the rights of individuals with mental health problems.

The IMHA’s attempt to be fully compliant with the UN-CRPD has led, at times, to vague language and opaque terminology on key topics. For example, there is ambiguity concerning the inter-relatedness of capacity, consent and the nominated representative. In addition, in an attempt to minimise restriction of rights, the IMHA may actually result in a greater level of coercion, owing especially to the opacity and insufficient review relating to nominated representatives, which are major concerns and will need careful and comprehensive review once the Act is implemented. These may be concordant with the UN-CRPD on paper but the outcome of the law and its effect on patients’ rights are more important than the theoretical principles affirmed by it. Realisation-focused mental health legislation and implementation programmes may do more to protect the health and rights of Indians with psychosocial disabilities than a pure focus on arrangement-focused legislation [61]. In other words, practice trumps theory every time.

Three groups are at particular potential risk: patients being treated in the community under an advanced directive, non-protesting patients, and long-term independent patients. The role of supported treatment in a community setting needs to be directly addressed. This may represent a less restrictive form of treatment in certain cases, but ambiguity in the area of advance directives may circumvent the proposed safeguards and potentially limit the rights of individuals concerned. Similarly, additional consideration should be given to non-protesting patients, although a supported admission framework may indeed prove the most appropriate means of facilitating admission in many such cases. The protection of long term voluntary patients is also an area of concern; the IMHA may reduce the numbers of individuals who remain in hospital as voluntary patients despite having high support needs (above).

Recognizing a patient’s capacity to give or withhold informed consent despite being a supported patient is an important step forward in the affirmation of the rights of individuals with mental illness. It also correctly identifies capacity as decision-specific. This idea could be incorporated into many laws concerning the rights of persons with disabilities and would do much to maximize the realization of their capacity and rights. The move away from a binary view of capacity, supported by some parts of the IMHA, is undoubtedly a positive one but an adequate review process must be retained.

Three important administrative issues arise which, if addressed, could further improve the new legislation. First, additional clarity could be added on the qualifications of individuals who can determine mental disorder. Second, other Indian legislation needs to be revised and updated to bring it in line with the new IMHA (e.g. the Medical Termination of Pregnancy Act, 1971). Third, and most importantly, consideration should be given to statutory collection of statistics about individuals receiving treatment, types of treatments being received, etc.

Overall, it is likely that India’s new mental health legislation will impact on more individuals than any other piece of mental health legislation in the world [62]. It is a carefully constructed document that addresses many of the needs of individuals with mental health problems. While clarification and change are certainly needed in specific areas, other countries revising their legislation would undoubtedly benefit from studying India’s constructive, pragmatic and enlightened approach to this matter.

## Abbreviations

BPAD: bi-polar affective disorder
CMHA: Central Mental Health Authority
DALY: disability-adjusted-life-years
ECT: electro convulsive therapy
IMHA: Indian Mental Healthcare Act
MHRB: Mental Health Review Board
RPDA: Rights of Persons with Disability Act
SMHA: State Mental Health Authorities
UN: United Nations
CRPD: Convention on the Rights of Persons with Disability
WHO: World Health Organization
WHO-RB: World Health Organization resource book on mental health, human rights and legislation

## Footnotes

1 RPDA 2016, section 34(1).

2 IMHA 2017, section 14(6).

3 IMHA 2017, section 98(1).

4 IMHA 2017, section 100(7).

5 IMHA 2017, section 94(1).

6 IMHA 2017, section 89 & 90.

7 IMHA 2017, section 89(1)(a).

8 IMHA 2017, section 89(1)(a).

9 IMHA 2017, section 89(1)(a).

10 IMHA 2017, sections 65 & 66.

11 IMHA 2107, sections 89(1)(b) & 90(2)(b).

12 IMHA 2017, section 89(9).

13 IMHA 2017, sections 89(10) & 90(14).

14 IMHA 2017, section 90(5).

15 IMHA 2017, section 90(4).

16 IMHA 2017, section 90(9).

17 IMHA 2017, section 89(13) & 90(15).

18 IMHA 2017, section 22.

19 IMHA 2017, section 86(2)(b).

20 IMHA 2017, section 89(1)(b).

21 IMHA 2017, section 87(12).

22 IMHA 2017, section 14(9).

23 IMHA 2017, section 81.

24 IMHA 2017, section 85.

25 IMHA 2017, sections 89(1)(a) & 90(2)(a).

26 IMHA 2017, sections 86(3), 89(1)(c) & 90(2)(c).

27 IMHA 2017, section 2.

28 IMHA 2017, section 21(1)(c).

29 IMHA 2017, sections 89(1)(a) & 90(2)(a).

30 IMHA 2017, sections 89(7), 90(12) & 94(1).

31 IMHA 2017, sections 5–13.

32 IMHA 2017, sections 89(8) & 90(13).

33 IMHA 2017, section 14(6).

34 IMHA 2017 section 2(1)(s).

35 IMHA 2017, section 3(1).

36 IMHA 2017, sections 5–13.

37 IMHA 2017, sections 14–17.

38 IMHA 2017, section 4.

39 IMHA 2017, section 4.

40 IMHA 2017, section 81(1).

41 RPDA 2016, section 14.

42 RPDA 2016, section 14(1).

43 RPDA 2016, section 14(3).

44 IMHA 2017, section 14.

45 IMHA 2017, section 17.

46 IMHA 2017, section 94(4).

47 IMHA 2107, section 114(1).

48 IMHA 2017, section 99.

49 IMHA 2017, section 94(1).

50 The Medical Termination of Pregnancy Act, 1971, section 3(2).

51 The Medical Termination of Pregnancy Act, 1971, section 3(4)(b).

52 The Medical Termination of Pregnancy Act, 1971, section 3(4)(a).

53 RPDA 2016, section 92(f).

54 IMHA 2017, section 94(1).

55 RPDA 2016, section 13(2).

56 RPDA 2016, section 14.

57 IMHA 2017, section 82.

58 IMHA 2017, sections 77(1), 89(10) & 90(14).

59 IMHA 2017, section 89(10).

60 IMHA 2017, sections 90(4–7).

61 IMHA 2017, section 83.

62 IMHA 2017, section 74(1).

63 IMHA 2017, section 43.

64 IMHA 2017, section 55.

65 IMHA 2017, section 67(1).

66 IMHA 2017, section 2(1)(y).

67 IMHA 2017, section 46(1) & 34(1).

68 IMHA 2017, section 46(1).

69 IMHA 2017, section 34(1).

70 IMHA 2017, sections 43(1)(b) &55(1)(b).

71 IMHA 2017, section 81(2).

72 IMHA 2017, section 82(3).

73 RPDA 2016, sections 66–71.

74 RPDA 2016, section 60–65.

75 IMHA 2017, sections 28, 43(1)(c), 55(1)(c), 77(1) & 82(1)(e).

76 IMHA 2017, section 14.

77 IMHA 2017, section 27.

78 IMHA 2017, section 87(3)(b).

79 IMHA 2017, section 17(a).

80 IMHA 2017, section 18(2), & 21.

81 RPDA 2016, section 4.

82 IMHA 2017, section 99.

83 IMHA 2017, section 100(1).

84 IMHA 2017, section 92.

85 IMHA 2017, section 102.

86 IMHA 2017, section 103(1).

87 IMHA 2017, section 103(6).

88 IMHA 2017, sections 102 & 105.

89 Indian Penal Code, section 84.

90 IMHA 2017, sections 102 & 105.

91 Code of Criminal Procedure, sections 328–335.

92 Code of Criminal Procedure, section 335(2).

93 IMHA 2017, section 95(1)(a).

94 IMHA 2017, sections 89(7) & 90(12).

95 IMHA 2017, section 95(2).

96 IMHA 2017, sections 18(2) & 21.

97 IMHA 2017, sections 5(3) & 81.

98 IMHA 2017, sections 86(3), 89(7) & 90(12).

99 IMHA 2017, section 14(1).

100 IMHA 2017, section 14(3).

101 IMHA 2017, section 14(6).

102 IMHA 2017, section 14(6).

103 IMHA 2017, section 14(7).

104 IMHA 2017, section 5(3).

105 IMHA 2017, section 14(9).

106 IMHA 2017, section 85(1).

107 IMHA 2017, section 2(1)(s).

108 IMHA 2017, section 115(2).

109 IMHA 2017, section 115.

110 IMHA 2017, section 18(1).

111 IMHA 2017, section 20.
